# Clinical decision making and risk appraisal using electronic risk assessment tools for cancer diagnosis: a qualitative study of GP experiences

**DOI:** 10.3399/BJGPO.2024.0243

**Published:** 2025-05-07

**Authors:** Alex Burns, Emily Fletcher, Elizabeth Shephard, Raff Calitri, Mark Tarrant, Adrian Mercer, William Hamilton, Sarah Dean

**Affiliations:** 1 University of Exeter Medical School, University of Exeter, Exeter, UK; 2 School of Psychology, University of Plymouth, Plymouth, UK; 3 ERICA Trial Patient and Public Involvement and Engagement Group, University of Exeter, Exeter, UK

**Keywords:** cancer diagnosis, neoplasms, primary health care, risk assessment

## Abstract

**Background:**

Electronic risk assessment tools (eRATs) are intended to improve early primary care cancer diagnosis. eRATs, which interrupt a consultation to suggest a possibility of a cancer diagnosis, could impact clinical appraisal and the experience of the consultation. This study explores this issue using data collected within the context of the Electronic RIsk-assessment for CAncer (ERICA) trial.

**Aim:**

To explore views and experiences of GPs who used the ERICA eRATs, how the tools impacted their perception of risk and diagnostic thinking, and how this was communicated to patients.

**Design & setting:**

Qualitative interviews with GPs from English general practices undertaking the ERICA trial.

**Method:**

Participants were purposefully sampled from practices participating in the intervention arm of the ERICA trial. Eighteen GPs undertook semi-structured interviews via Microsoft Teams. Thematic analysis was used to explore their perspectives of the impact of the eRATs on consultations, diagnostic thinking related to cancer and other conditions, and how this information is communicated to patients.

**Results:**

The following three themes were developed: 1) the armoury, whereby eRATs were perceived as ‘additional armour’, offering a layer of protection against missing a cancer diagnosis, the defence coming at a cost of anxiety and complexity of consultation; 2) ‘three heads’ making a decision. eRATs were seen as another actor in the consultation, separate from clinician and patient, and challenging GP autonomy; and 3) for whom is the eRAT output intended? GPs were conflicted about whether the numerical eRAT outputs were helpful when communicating with patients.

**Conclusion:**

eRATs are appreciated as a defence against missing a cancer diagnosis. This defence comes at a cost and challenges GPs’ freedom in communication and decision making.

## How this fits in

The use of electronic risk assessment tools (eRATs), which interrupt the clinical consultation, have been encouraged to support early diagnoses of significant, time-critical conditions. Little real-world evidence of their effectiveness or how they impact the clinical consultation exists. Using GP interview data from a large clinical trial (the Electronic RIsk-assessment for CAncer [ERICA] trial), this study explored how eRATs for cancer influenced clinical reasoning. Although the principle of eRATs was appreciated, the complexity of the consultation increased with GPs reporting a loss of clinician freedom both in terms of communication with patients and decision making.

## Introduction

Policies encouraging improved cancer care in the UK started after studies indicated a worse cancer survival rate compared with other high-income countries.^1^ This adverse trend has persisted.^2–4^ Given early stage at diagnosis is associated with improved survival,^5^ early primary care diagnosis has been targeted as a potential solution.^6^ In the UK, the urgent suspected cancer (USC) pathway (previously known as the ‘2-week wait’ pathway) is the route to early diagnosis from primary care, and while there is evidence that rapid diagnosis is achieved through this pathway,^7^ there is substantial variation in its use.^8^

One suggested method of increasing appropriate use of the USC pathway is via eRATs.^9^ These computer-based tools predict cancer by aggregating their relevant computer-coded symptoms, test results, and demographic data already present in the GP’s clinical system.^10^ They apply population-based risk estimates to the individual patient. Although there is no clear evidence of their effectiveness in primary care,^11–13^ their use is now encouraged in the UK.^14^

Primary care diagnosis presents challenges owing to the variable, imperfect, and subjective nature of the main input: patient history and examination. The pre-test probability of pathology in primary care is comparatively lower than in other healthcare contexts,^15^ meaning GPs need to navigate risk and embrace uncertainty in the diagnostic process.^16^ The traditional diagnostic paradigm involves a patient explaining their symptoms to a clinician, who then formulates a differential diagnosis, which is refined via examination, simple observations, and, if required, testing.^17,18^ It is at this point that previous risk assessment tools (electronic or otherwise) have been designed to work.^10^ In this situation a clinician must first consider that the possibility of cancer is high enough to warrant using a risk tool. This choice, of deciding if the tool is appropriate, is removed with some eRATs, where an on-screen prompt automatically interrupts the consultation with cancer risk information. It could disrupt the consultation dynamic, divert consultations from being patient led, or cause overtesting for cancer.

Previous qualitative studies examining how eRATs impact decision making have mainly used simulated clinics or prototype systems. Some studies have shown that they can help highlight possible cancer, or other serious diagnoses,^19^ and that, in some cases, the risk identified by the eRAT is greater than that perceived by the clinician.^20,21^ The time it takes to use eRATs has been a barrier to their use.^21–23^ Clinicians may mistrust the underlying algorithms and perceive them as fear-inducing,^24,25^ and have a preference for their own clinical judgement or intuition.^26,27^ These issues may contribute to the low uptake of eRATs in primary care.^28^

A novel set of eRATs for cancer (the ERICA eRATs) are algorithms based on established risk scores^29–34^ to help identify lung, colorectal, oesophago-gastric, bladder, kidney, and ovarian cancers. These eRATs operate through two mechanisms. First, an on-screen alert shows a numerical percentage risk when a patient’s risk of any of the six cancers reaches ≥2% (National Institute for Health and Care Excellence [NICE] threshold for referral is ≥3% risk,^35^ but a 2% trigger is intended to let clinicians explore further symptoms if appropriate). Second, clinicians can actively use a ‘symptom checker’ to review relevant symptoms, adding patient-specific symptoms and recalculating cancer risk accordingly. The ERICA eRATs are undergoing assessment of their clinical and cost-effectiveness as part of a large, ongoing pragmatic cluster-randomised control trial.^36^ At the time of its initiation, the ERICA trial was the first large trial to investigate effectiveness of eRATs with automatic on-screen prompts for a high-stakes diagnosis in a real-world setting. Understanding the impact of eRATs on GPs’ clinical reasoning, within this real-world clinical context, will contribute to an assessment of their potential utility.

The aim of this study was to explore views and experiences of GPs who used the ERICA eRATs, how the tool impacted their perception of risk and diagnostic thinking, and how this was communicated to patients.

## Method

A qualitative interview nested study was conducted as part of the ERICA trial.^36^ The study had the following three separate objectives: to explore 1) the eRATs’ implementation process; 2) GP experiences of eRATs on risk appraisal and clinical decision making (reported here); and 3) the effect of eRATs on workload and workflow. The interview guide (see Supplementary Appendix S1) included questions related to all three objectives. This approach balanced methodological considerations with pragmatism and resources. It enabled researchers to collect data from a comparable number of clinicians, as recruited in similar qualitative studies.^25,27^ We present these findings separate to the two other study objectives, as these findings are less specific to the ERICA intervention implementation and workload issues. Instead, these findings may offer insight into clinician–patient–eRAT interactions more widely.

GPs using the eRAT software in the intervention arm of ERICA were invited to interviews. Email invitations were sent in batches of 10, with a reminder sent 2–3 weeks later. Recruitment was purposeful, targeting a sample framework to include both main IT systems used in UK primary care (EMIS and SystmOne) and across a range of USC pathway use. The interview schedule and consent forms were emailed to the responders, and an interview time arranged. A 30-minute face-to-face or online interview via Microsoft Teams was offered. Interviews were conducted by AB and EF. Participants were given a £50 voucher as a token of appreciation.

Interviews were transcribed by a third party. Transcripts were read and coded inductively by AB, following the process of thematic analysis.^37^ Analysis was approached from a position of critical realism,^38^ assuming that a cancer diagnosis exists independent of human observation, but there are perceptual layers, such as symptoms, signs, tests, and eRAT scores, which are an imperfect representation of that reality. Information power^39^ was considered iteratively after each interview and discussed with the research team.

NVivo software was used. Initial codes were broad, capturing any meaning associated with diagnostic decision making, risk, or uncertainty. Coded segments were grouped into an initial descriptive code framework. Codes and their groupings were discussed within the research team. The descriptive coding framework evolved throughout the process of coding, with particular code areas expanding as more data were added. Once all transcripts had been coded, a second researcher (EF) independently coded four transcripts using the coding framework. This was to explore if further meaning could be extracted from the data, or if any data related to the descriptive codes had been overlooked.

Descriptive codes were explored for overlap, or use of language and metaphor, which indicated a higher level or more abstract meaning. From this, initial shared ideas or emergent themes were discussed between AB and the research team. Analysis then developed into the reported themes.

The ERICA trial had an established patient and public involvement and engagement (PPIE) group from the outset. The group have input into key aspects of trial design including development of the interview topic guides. They have also been involved in trial oversight with PPIE representation at trial group meetings and have contributed to making sense of study findings; the emerging themes in this study were reviewed and discussed with the PPIE group before finalising.

## Results

Eighteen GPs were recruited from a total of 70 invitations. Interviewees were not known to AB or EF. Characteristics of the 18 participants are shown on Table 1. Average length of interview was about 32 minutes. Further supporting quotes are displayed in Supplementary Table S1.

**Table 1.** GP and practice participant characteristics

Characteristics of participants and their practices		Interviewees, *n*
Practice clinical system	TPP SystmOne	8
	EMIS	10
Practice urgent suspected cancer referral ratio^a^	Low (13.57–85.16)	3
	Medium (85.17–107.30)	6
	High (107.31–265.87)	9
Practice list size	Small or medium (<3500–8000)	6
	Large (>8000)	12
Practice deprivation rating	1–5 (more deprived)	12
	6–10 (less deprived)	6
Local Clinical Research Network region	East of England	2
	North East and North Cumbria	1
	North West Coast	1
	Northern Thames	1
	South London	1
	South West Peninsula	8
	Wessex	2
	West of England	2
Sex of participant	Male	7
	Female	11
Age of participant, years	30–40	3
	41–50	2
	51–60	5
	Not known	8
Experience as clinician, years	0–10	4
	11–20	2
	>20	4
	Not known	8

^a^This ratio is the number of urgent suspected cancer referrals observed in the registered population divided by the number ‘expected’, based on the practice’s age–sex specific population and the age–sex specific rates for England. The unit is ‘per 100 patients’, thus the ratio for a practice at the numerical medium is 100.*^55^*

The following three themes were identified: the armoury; ‘three heads’ making a decision; and for whom is the eRAT output intended?

### Theme 1: The armoury

This theme considers the risks and benefits of the eRATs and how they provide an additional layer of protection against missed diagnoses. This protection is for the patient, but also for the clinician. Participants recognised the principles on which eRATs work and are supportive of their objectives. It is *’part of our working armoury’* (GP15), which *’spots things that you miss, because you’re thinking about so many other things’* (GP09).

Its use in practice is more complicated. The armour has a cost. Armour is heavy and restricts movement: the eRATs hinder a clinician’s freedom of movement and adds to complexity of the consultation.

Participants were ambivalent about whether the costs were worth the benefits, and felt that over time this could lead to alert fatigue:

*’... your resistance to them would gradually go down and down and down, and after a while you’d be like, what? Not really noticing.’* (GP15)

There was a perception that this eRAT armour was often redundant: *’it’s really very difficult to sort out the actual signal from the noise’* (GP15). *’Noise’* here could be because the patient had already undergone, or was waiting for, investigations for the cancer that an eRAT had flagged:

*’I can ignore that prompt because I’m satisfied that those features that are triggering it have already been reviewed, actioned, or referred … ‘* (GP02)

’*Noise*’ also occurred when the component triggers were better explained by an alternative plausible narrative:

*’I feel like I can justify that ... it reduces my concern.’* (GP16)

Clinicians felt that the eRAT might be more helpful for GPs trained before the 2015 NICE guidelines,^35^ or those from practices with poor continuity of care.

Participants’ understanding of the principles of the eRAT algorithms also led to scepticism about its protection. Because the eRATs works from symptom coding, the algorithms are only as good as the input provided by the clinician, which contained *’massive variability. Completely unreliable’* (GP05). Clinicians felt that the symptoms they coded were likely ones they already considered significant:

*’Symptoms such as diarrhoea. There’s no way on God’s earth we are coding that regularly … ‘* (GP10)

This leads to a circular paradox, predisposing towards warnings of cancer risks that have already been addressed:

*’I guess the problem about it is that when I’m … if I’m coding things which have a significant impact on a … on a possible diagnosis, I’m actually already working towards that diagnosis.’* (GP15)

### Theme 2: ‘Three heads’ making a decision

In this theme the eRATs introduce another actor into the consultation, changing the locus of decision making, and challenging clinical autonomy.

The traditional clinical consultation model involves a clinician and a patient (or someone acting as their agent, such as a caregiver or relative). In a consultation with an eRAT, participants felt it was a third actor, with its own priorities and agency:

*’I think you have to be having almost three heads on suddenly. Thinking about, what could the computer be telling me? What can the patient be telling me and what am I thinking?’* (GP09)

This increased the complexity of the consultation, as well as adding uncertainty about whose priorities were most important and about *’where does the responsibility lie?’* (GP09).

Most participants felt that the patient’s agenda should have priority over other considerations, but that the eRATs could threaten this:

*’There’s so many things … which take you away from why the patient has actually come to see you … and it’s another thing. We’ve kind of had enough of that.’* (GP14)

Some clinicians felt their professional identity was threatened by the eRATs. They felt the eRATs undermined the usefulness of clinical judgement and shifted the diagnostic process into the administrative realm, away from their medical expertise:

*’... if we just turn it into, you know, computer says yes, computer says no, at this level, what does this do to … you know, to our patients?’* (GP15)

### Theme 3: For whom is the eRAT output intended?

The final theme raises fundamental issues about communicating and understanding of numerical risk, and patient autonomy relating to these points. Participants raised the question as to who should be informed about the risk information. They felt conflicted when discussing the numerical results of the eRATs with patients. There was a recognition that in some cases, it added transparency to the discussion of risk and could be used to share diagnostic thinking and responsibility. Participants reflected on the 3% risk threshold for USC referrals, considering the balance between motivating patients to attend their appointments and reassuring them about the low risk of having cancer.

A more common perspective was that numerical risk information was not appropriate for patients:

*’I wouldn’t normally converse in percentage terms with patients.’* (GP01)

Participants felt that patient understanding of numerical risk was poor and could lead to anxiety while restricting the clinical freedom of the clinician:

*’I feel patients get very hung up on numbers sometimes, so I try not to … get myself nailed down too much*.*’* (GP13)

Some participants felt that there was an ethical imperative to share the numerical risk with the patient, as it is *’their information ... it’s their risk’* (GP16). Others, in contrast, thought that this information was intended for the clinician alone:

*’… like, this is for me to use, it’s not for the patient to use … ‘* (GP09)

Participants were more comfortable sharing the individual symptoms and risk factors than the resulting numerical risk.

Participants felt that the categorical nature of clinical coding was inappropriate for subjective symptoms, and translating symptoms in diagnosis required clinical judgement.

## Discussion

### Summary

Participants appreciated the intention of the eRATs as a defence against missing a cancer diagnosis. In practice, the designed attributes of high sensitivity and automaticity of triggering create the issues that clinicians experience in their use. Initially, there was an impression that the eRATs confirmed rather than suggested the diagnosis, then a recognition that it increased consultation complexity and challenged the autonomy of clinicians in their communication and freedom of clinical reasoning.

### Strengths and limitations

This study’s primary strength lies in its utilisation of data from clinicians actively using eRATs in clinical practice, from diverse practice settings, encompassing both high and low USC referral rates. Although sample size was pre-specified by the design of the ERICA trial, participants were articulate professionals, and the dialogue collected was thematically rich. Evidence of recurrent themes and patterns across participants occurred early in data analysis, supporting cross-case analysis and confidence to the adequacy of the sample’s ‘information power’.^39^

Potential limitations include the partial self-selection of participants. We could only interview ERICA users, which was a potential bias towards ‘early adopters’^40^ or individuals who prioritise cancer diagnosis. Clinicians unengaged with the eRATs, a potential cause of any null outcome in the ERICA trial, are not represented here. It also necessarily involved participants who had been recently introduced to eRATs. A more prolonged exposure to any alert system may change a clinician’s interaction with that system through assimilation into normal routine.

The corresponding author (AB) can be considered an ‘insider researcher’, as a practising GP.^41^ This was made known at recruitment and had a positive impact on uptake. It may have improved data collection, by fostering trust and deeper understanding with participants at interview but could potentially introduce bias through personal assumptions or shared experiences.^41,42^

### Comparison with existing literature

The eRATs’ model is supported by studies showing that diagnostic suggestions introduced early into the consultation are more effective in improving diagnostic accuracy,^43^ perhaps by avoiding anchoring bias (over-reliance on initial information or working diagnosis) and egocentric advice discounting (dismissing advice that conflicts with one’s own beliefs).^44^ However, theme 3 explored clinicians’ misgivings with clinical coding, and the use of subjective inputs to create a numerical risk score. Clinical reasoning can be described as a progression from subjective inputs through to a categorical diagnosis.^45^ eRATs, and their insertion of objective information early in a consultation, could disrupt this progression and challenge a clinician’s autonomy of selecting objective tests or other information (such as scores) that they deem necessary. This challenge to a clinician’s ‘epistemic authority’ has been previously described.^46^ It may lead to disengagement with the eRAT, or clinicians considering apparently objective information as subjective,^47^ undermining the purpose of the eRAT.

Theme 1 suggests that clinicians discount eRATs if they feel there is a more likely alternative diagnosis. The underlying logic of diagnosis is that evidence raising the probability of one disease reduces the probability of all remaining possibilities.^48^ Although eRATs provide a predictive population-based risk, there is less evidence of how alternative diagnoses should influence USC referrals. As most patients at the NICE referral threshold of 3% will not have cancer whether referred or not, clinicians do not have feedback with which to calibrate their decisions, an important part of improving diagnostic thinking.^49^

One participant used the terminology of signal detection theory^50^ (and others employed similar language) to describe their interaction with the eRAT, referring to the ‘signal-to-noise ratio’. If eRAT triggers are perceived to offer additional noise (and thus workload and cognitive burden) without discernible signal, then this may cause clinicians to disengage: a process described as ‘alert fatigue’.^51^

Theme 3 explored views about sharing the risk output of the eRAT with the patient. Shared decision making in diagnosis, where clinicians discuss and decide with a patient how to proceed, is a laudable aim.^52^ When discussions regarding further risk scores or testing take place before the test is performed, the ethical duty of sharing the results with a patient is clear.^53^ When the diagnostic information has not been previously discussed, the clinician may feel less obliged to share it. In this study, participants weighed their ethical duty to communicate the eRAT against the increased complexity, anxiety, and uncertainty created.

Although eRAT use in cancer diagnosis is currently encouraged, there is a lack of evidence for their effectiveness and the main ERICA trial aims to address this gap. The findings discussed here can be more generally applied. They highlight the challenges posed by an eRAT to the diagnostic process, including increased consultation complexity, difficulties in communicating numerical risk, and concerns about perceived clinical autonomy. In considering their use, GPs need to weigh up the putative benefits against the findings presented.

### Implications for research

The use of digital technology in medicine is increasing,^54^ and research into clinician and patient interaction with computers is needed to maximise their potential. Further research is needed into how computer-generated risk information is received and processed by clinicians. It may be that new models of clinical reasoning are required, which explicitly incorporates computer-generated risk scores and diagnostic suggestions at the outset, when a patient presents with a complaint.

## Data

The dataset relied on in this article is available from the corresponding author on reasonable request.
